# The Equivalents Are Coming! Developing a Shared Nomenclature for Radiopharmaceutical Therapy of Neuroendocrine Tumors, Prostate Cancer, and Beyond

**DOI:** 10.2967/jnumed.126.272026

**Published:** 2026-07

**Authors:** Aman Chauhan, Andrew E. Hendifar, Ghassan El-Haddad, Heloisa P. Soares, Ken Herrmann

**Affiliations:** 1 School of Medicine, University of California, San Francisco, San Francisco, California;; 2 Division of Hematology-Oncology, Department of Internal Medicine, Cedars-Sinai Medical Center, Los Angeles, California;; 3 Department of Diagnostic Imaging and Interventional Radiology, H. Lee Moffitt Cancer Center and Research Institute, Tampa, Florida;; 4 Department of Internal Medicine, Division of Oncology, Huntsman Cancer Institute at University of Utah Health Care, Salt Lake City, Utah;; 5 Department of Nuclear Medicine, University of Duisburg-Essen, Essen, Germany; and; 6 German Cancer Consortium, University Hospital Essen, Essen, Germany

**Keywords:** radiopharmaceutical therapy, regulatory, lexicon, oncology, 505(b)(2)

## Abstract

As radiopharmaceutical therapy (RPT) expands beyond neuroendocrine tumors and prostate cancer, it is important to understand the U.S. Food and Drug Administration (FDA) approval process and to establish a shared vocabulary for effective communication. The 505(b)(2) New Drug Application is a well-established mechanism for FDA approval of nuclear medicine products. It is an application for drugs that are similar to an approved product, supported by published literature and studies conducted on similar drugs, and bridging studies demonstrating the scientific relevance of the data to the product. This streamlined approval pathway can encourage development of more treatment options, facilitating greater patient access and improved resilience to supply chain disruptions. The evolving RPT landscape necessitates new nomenclature. “Radioligand equivalent” refers to an RPT approved through the 505(b)(2) pathway, distinct from a generic RPT approved through an Abbreviated New Drug Application. Establishing shared terminology supports clear multidisciplinary communication and fosters informed treatment selection.

## Visual Abstract

**Figure:**
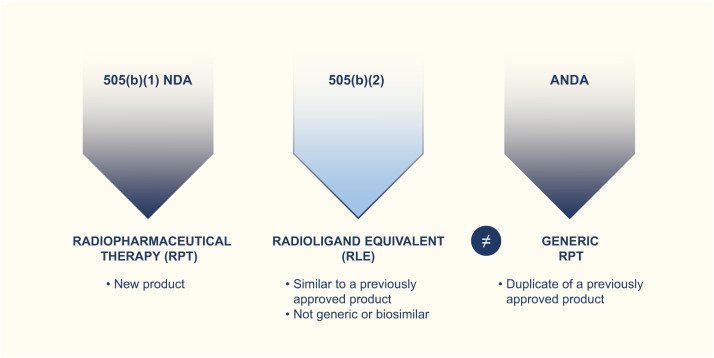

As radiopharmaceutical therapy (RPT) expands beyond neuroendocrine tumors (NETs) and prostate cancer, nuclear medicine physicians must become familiar with multiple regulatory designations influencing access and evidence. RPT is an inherently cross-disciplinary treatment involving nuclear medicine physicians, radiation oncologists, oncologists, and relevant medical specialists such as endocrinologists, urologists, pathologists, and geneticists (1). With the evolving RPT landscape, it is important to understand the U.S. Food and Drug Administration (FDA) approval process and establish a shared vocabulary for effective communication among multidisciplinary teams.

## THE 505(b)(2) PATHWAY

Regulatory pathways are commonly referred to by their designated sections of the *Federal Food, Drug, and Cosmetic (FD&C)* Act. Most clinicians are likely familiar with 2 key mechanisms: the stand-alone 505(b)(1) New Drug Application (NDA) for new products and the 505(j) Abbreviated New Drug Application (ANDA) for generic products. Many may be less familiar with the 505(b)(2) NDA pathway, a hybrid of these 2 options. It has become a mainstay of new drug approvals because it streamlines drug development, with the potential for more treatment options, greater patient accessibility, and improved resilience to supply chain disruptions.

505(b)(2) is a pathway for new drugs that are similar to an existing product, with some differences in characteristics permitted. There are several types of 505(b)(2) submissions. An analysis of FDA data from 2012 to 2016 found that most drugs approved through the 505(b)(2) pathway were a new formulation or new manufacturer (43%) or new dosage form (29%). Less common submission types were for a new combination (13%), a drug already marketed without an approved NDA (8%), a new molecular entity (2%), and a new active ingredient (1%) (2).

Products approved through the 505(b)(2) pathway are not considered generics or biosimilars. The term *biosimilar* is reserved for biologic medications (i.e., products made from natural living sources) by the FDA (3). Generics are duplicates of a previously approved product and are approved through the ANDA 505(j) pathway (4). Generics are required to be therapeutically and pharmaceutically equivalent to a reference drug and rely on the FDA’s previous findings of safety and effectiveness of the listed drug. By contrast, the 505(b)(2) pathway allows for differences in the indication, formulation, dosage form, dosing regimen, route of administration, strength, and active ingredient and requires full reports of investigations of safety and effectiveness (4,5).

## 505(b)(2) SUPPORTING EVIDENCE

A key difference between the 505(b)(2) pathway and a stand-alone 505(b)(1) NDA is the type of supporting information required in the submission for FDA approval. The FDA defines a 505(b)(2) application as “an NDA submitted under section 505(b)(1) and approved under section 505(c) of the FD&C Act that contains full reports of investigations of safety and effectiveness, where at least some of the information required for approval comes from studies not conducted by or for the applicant and for which the applicant has not obtained a right of reference or use” (4). A stand-alone NDA, on the other hand, is defined as “an application submitted under section 505(b)(1) and approved under section 505(c) of the FD&C Act that contains full reports of investigations of safety and effectiveness that were conducted by or for the applicant or for which the applicant has a right of reference or use” (4).

This means that, unlike a product submitted under a stand-alone NDA, a 505(b)(2) product could be approved based on data from published literature or efficacy and safety studies of relevant FDA-approved drugs (5). To demonstrate that data from trials of other products are relevant and appropriate, applicants must establish a scientific bridge to each listed drug or published study. The type of bridging data required depends on what characteristics the new product shares with the listed drug and could include in vitro, in vivo, or in-human studies showing that there are no clinically meaningful differences in pharmacology, efficacy, and safety (4).

For example, methylphenidate hydrochloride extended release (NDA 209311) is a capsule taken in the evening to treat attention-deficit/hyperactivity disorder. The 505(b)(2) application was supported by the label of an approved formulation of methylphenidate HCl, published literature, clinical trials, a supporting trial of a different formulation, and pharmacokinetic studies demonstrating bioequivalence to the listed drug (6).

Selecting an appropriate treatment requires assessing a complex range of patient characteristics and clinical evidence of safety and efficacy. Clinicians considering treatment options that were approved through the 505(b)(2) pathway should be aware that patient selection is supported by a rigorous body of evidence that may include published literature, studies conducted on similar drugs, and bridging studies.

## 505(b)(2) IN NUCLEAR MEDICINE

505(b)(2) is a well-established mechanism for FDA drug review, including nuclear medicine products. We conducted a retrospective analysis of NDA approvals over the last 5 y, using data from the FDA’s Center for Drug Evaluation and Research for approvals between January 1, 2020, and December 31, 2024 (7). A total of 510 NDAs were approved during that period, and 55% of those NDAs were approved through the 505(b)(2) pathway. In the analysis, nuclear medicine products approved through the 505(b)(2) pathway were all imaging agents; no RPTs were identified. Examples include agents that expanded access for patients with NETs and prostate cancer: ^64^Cu-DOTATATE injection, kit for the preparation of ^68^Ga-DOTATATE injection, and kit for the preparation of ^68^Ga-gozetotide injection (Table 1) (7). These are distinct from generic radiopharmaceuticals approved through the ANDA pathway, for example, the diagnostic agent ^18^F-FDG (8).

**TABLE 1.** Examples of Nuclear Imaging Products Approved Through the 505(b)(2) Pathway

Product (7)	505(b)(2) approval year (7)	505(b)(2) submission type (7)	Indication
Kit for the preparation of ^68^Ga-DOTATATE injection, for intravenous use	2016	New molecular entity	Diagnostic PET imaging of NETs (9)
^64^Cu-DOTATATE injection	2020	New molecular entity	Diagnostic PET imaging of NETs (10)
Kit for the preparation of ^68^Ga-gozetotide injection, for intravenous use	2022	New active ingredient	Diagnostic PET imaging of prostate cancer (11)
Kit for the preparation of ^99m^Tc-succimer injection, for intravenous use	2022	New formulation or new manufacturer	Diagnostic scintigraphic evaluation of renal parenchymal disorders (12)

## RPT NOMENCLATURE

The RPT landscape is expanding, with multiple companies developing new products and indications. Given the multidisciplinary nature of RPT, it is important to use a consistent shared lexicon to discuss treatment options. The 505(b)(2) NDA is a widely used approval pathway for nuclear medicine products. Products approved through the 505(b)(2) pathway may be marketed with brand names rather than generic names, and the label and reimbursement codes may differ from the listed drug. Currently, there are no adequate terms to distinguish between RPT products approved through the NDA 505(b)(1), ANDA, and NDA 505(b)(2) pathways.

Being able to rapidly identify these products would help clinicians recognize the types of data available to inform their treatment decisions. The names for each type of product should be simple, self-explanatory, and easy to remember. We propose the term *radioligand equivalent* (RLE) for RPTs approved through the 505(b)(2) pathway that are similar or equivalent to a previously approved RPT. We propose *generic RPT* for RPTs approved through the ANDA pathway (Table 2). For example, an RLE for NETs could have the same somatostatin receptor ligand, chelator, and radionuclide as a previously approved RPT but differ in the production route, leading to a non-carrier-added rather than a carrier-added product, with different waste handling requirements.

**TABLE 2.** RPT Lexicon That Distinguishes Between FDA Approval Mechanisms

RPT term	Definition
RPT	RPT approved through the stand-alone NDA 505(b)(1) pathway
RLE	RPT approved through the 505(b)(2) pathway that is similar to an FDA-approved product
Generic RPT	RPT approved through the ANDA pathway that is a duplicate of an FDA-approved product

Related terms	FDA definition
Bioequivalents	Products with no significant difference in the rate and extent of absorption when they are administered at the same molar dose of the therapeutic ingredient under similar experimental conditions either in a single dose or multiple doses (4)
Pharmaceutical equivalents	Products with the same active ingredient(s), dosage form, route of administration, and strength (4)
Therapeutic equivalents	Approved drug products that are pharmaceutical equivalents for which bioequivalence has been demonstrated and that can be expected to have the same clinical effect and safety profile when administered to patients under the conditions specified in the labeling (4)
Biosimilar	Biologic product (i.e., a medication made from natural and living sources) that is highly similar to an already-approved biologic product (3)

Table 2 summarizes some other common terms used to describe products that are similar to each other. It is important to be aware that these terms have specific meanings defined by the FDA. Given their specialized use in the regulatory context, they are not appropriate for distinguishing between RPTs.

## CONCLUSION

The 505(b)(2) pathway is a well-established mechanism for FDA approval of nuclear medicine products and related therapies. 505(b)(2) products are approved on the basis of efficacy and safety, demonstrated through a rigorous body of evidence drawn from published literature, studies conducted on similar drugs, and bridging studies demonstrating the scientific relevance of the data to the product (4,5). This streamlined process can facilitate the development of more treatment options, leading to greater access and improved resilience to supply chain disruptions.

As the RPT field advances, adopting a shared, unambiguous nomenclature (RPT, RLE, and generic RPT) will promote transparency, facilitate multidisciplinary communication, and support evidence-based treatment selection.

## DISCLOSURE

Aman Chauhan has received grants or contracts from Seneca Therapeutics, BMS, and Clovis; consulting fees from Novartis, Crinetics (steering committee member), Curium, Lantheus, Exelixis (steering committee member), Ipsen, TerSera, Lexicon, Sanofi, Seneca Therapeutics, ITM, and Boehringer Ingelheim (steering committee member); and is on the board of directors for NANETS. Ken Herrmann has received consulting fees from Advanced Accelerator Applications, a Novartis company, Actithera, Amgen, AstraZeneca, Bain Capital, Bayer, Bicycle Therapeutics, Boston Scientific, Convergent, Curium, Debiopharm, EcoR1, Full Life, Fusion, GE HealthCare, Immedica, Isotopen Technologien München, Janssen, Merck, MSD, Molecular Partners, NVision, POINT Biopharma, Pentixapharm, Pfizer, Radiopharm Theranostics, Rhine Pharma, Siemens Healthineers, SOFIE Biosciences, Telix, Theragnostics, and Y-mAbs; receives research grants from Advanced Accelerator Applications, a Novartis company, Boston Scientific, and Janssen; and has stock or other ownership interests with AdvanCell, Aktis Oncology, Bicycle Therapeutics, Convergent, NVision, Perspective Therapeutics, SOFIE Biosciences, and Yellowbird Diagnostics. Heloisa Soares has received consulting fees from Curium, Ipsen, ITM, TerSera, Novartis, Exelixis, and Sanofi. Andrew Hendifar is a consultant for Novartis, Ipsen, Valar Labs, PANCAN, Faraday Pharmaceuticals, Amgen, Regeneron, Exelexis, Alcresta Therapeutics, Pfizer, Curium Pharma, and Merck and has received research funding from Ipsen, institutional research funding from NGM Biopharmaceuticals; travel fees from Halozyme; and other compensation from RayzeBio. Ghassan El-Haddad is a consultant for Bayer HealthCare, BD, Curium, ITM Radiopharma, Johnson and Johnson, Lantheus, and Novartis. This work received medical writing support from Relevate Health Group, LLC, in accordance with Good Publication Practice, funded by Curium US LLC, Maryland Heights, MO, USA. No other potential conflict of interest relevant to this article was reported.

## Notes

KEY POINTS **QUESTION:** What is an appropriate lexicon to distinguish between RPTs approved by the FDA through the stand-alone NDA, ANDA, and 505(b)(2) pathways? **PERTINENT FINDINGS:** An RLE refers to an RPT approved through the 505(b)(2) pathway and is distinct from a generic RPT approved through the ANDA pathway. **IMPLICATIONS FOR PATIENT CARE:** Clear, unambiguous nomenclature facilitates multidisciplinary communication and enhances confidence in treatment selection for patients considering RPT.
